# Some Symptoms of Sycosis

**Published:** 1892-10

**Authors:** Geo. H. Clark

**Affiliations:** Germantown, Philadelphia


### SOME SYMPTOMS OF SYCOSIS.

Geo. H. Clark, M. D., Germantown, Philadelphia.

(Bureau of Clinical Medicine, I. H. A.)

To know what can be known of the peculiar manifestations of the various bodily constitutions, made more plain by Hahnemann's genius after years of keen observation in that he pointed out the origin of all diseases, will enable one to treat any given case of disease more intelligently.

There are many practitioners of Homœopathy who, while successful in curing patients, have no idea of the underlying principles by which cure is effected.

There is but little that can be known regarding the action of remedies. We can only know that adherence to the homœopathic law will bring success in every curable case, and relief, greater than afforded by any other practice, to the incurable.

Not only more knowingly but more easily can we arrive at the correct remedy if we give more attention to the generic disease-form as elaborated by Hahnemann in his so admirably worked-out psora, sycosis, and syphilis.

Of the truth contained in Hahnemann's observations we can easily convince ourselves and others if we take but psora, and give heed to his teachings by observations which each one can make for himself.

In another place I have called attention to the observations of Dr. Reuter, of Nuremberg, in respect to psora. "He found," says Granvogl, "stages, so to speak, like those which mark acute diseases, in the various forms of the reciprocal action of this poison with the organism."

This is the succession of stages in diseases arising from psora, provided that up to the last stage no medical aid had been sought:

"1, Gastroses; 2, catarrhs; 3, hemorrhoids; 4, sweat of the feet; 5, hoarseness; 6, headache and toothache; 7, diseases of the eyes; 8, diseases of the ears; 9, prurigo of the trunk, furunculosis; 10, swelling of the cervical glands; 11, rheumatismus; 12, swelling of the axillary glands.

"His experience indicated to him an aggravation of the general constitutional status, if after a disease from among those named under these numbers had passed by another of a higher number makes its appearance."

Here is an example of what may be learned by close observation. With this before us it will require but slight observation on our part to add the various affections due to the sycotic poison.

Although psora, or rather its improper treatment, can present so long a list of formidable affections, sycosis does not lag far behind with a schedule of ailments that can properly be pronounced by him who has met and recognized them as even more powerful for causing suffering.

Beginning with the sycotic form of gonorrhœa, upon which so many, particularly old-school physicians, and through them their much-to-be-pitied victims, look as a mere trifle, improperly treated by injections, and thereby suppressed, it may remain latent in the system for many years.

If it ended here we should be under no obligations to give the subject much thought. But when we know how it is transmitted, and of the sufferings entailed upon the innocent, even unto the third and fourth generations, it is incumbent upon us as conscientious physicians, followers of Hahnemann, and as honest men, to raise our voices against a practice that is responsible for so much distress, both mental and physical.

There are no more constant symptoms of sycosis than those of the mind. Indeed, the anguish caused by this poison makes it

> * * * "A monster of so frightful mien
> As to be hated needs but to be seen."

For a number of years I have been able to recognize the presence of this poison by the unusual anxiety which is always present. In more advanced cases anxiety gives place to anguish. Anxiety is disquietude, care, solicitude, uneasiness, concern. Anguish is pain, pang, distress, suffering, woe, torture, torment, agony, grief, sorrow, discomfort.

Compare these synonyms with the mental symptoms of remedies in whose pathogeneses they are found and you may put down those remedies as anti-sycotics.

The physical symptoms characteristic of this miasm are of such a character that they usually aggravate the mental state in both man and woman.

Both may to all appearance be in excellent health, so far as external signs may be noted.

But meet with some known, unmistakable symptom of sycosis, and then ask the patient—or you can confidently tell him, if you please—about the mental state. You will find anguish a constant symptom.

You will also find inability for consecutive thought; fear of misspelling simple words, fear of being able to write even a short note that can be understood; great irritability, particularly in men; fear of associating with strangers, and of going into a crowd; avoidance of any one outside the immediate family, and even repugnance to his own children.

If any acute affection appear, it will be dominated by the peculiar mental state, no matter how slight the ailment.

In short, the intellectually brilliant are mere mental wrecks.

The most characteristic physical sign of sycosis is great muscular debility. Although a man may have the best muscular development, he is incapable of the least exertion without fatigue, this more particularly after his chronic ailments have been treated allopathically. Even a short walk tires him so much that he is glad to rest. This, reacting on the mind, through realizing what he now is, in comparison to what he was before the affection was manifest, aggravates the mental state.

And so it will go on month after month, year after year, until he seeks aid of the Hahnemannian.

And what a picture he now presents!

It will require more patience, more study, and a greater expenditure of energy on the part of the conscientious physician than any condition he attempts to treat—particularly when complicated with psora.

Of physical pain the man may suffer but little. It is the woman who has added to the mental state bodily pain. In woman the most marked of the chronic symptoms of sycosis will be found in the ovario-uterine sphere, and reflexly upon the brain and spinal cord. Dysmenorrhœa of the most severe character is prominent. It is marked in this: the pain of dysmenorrhœa of any ordinary nature usually precedes the flow, and disappears as the flow comes on. In sycotic dysmenorrhœa the pain is agonizing, and may continue to the end of the flow, and there is pain before and after the flow. Before, during, and after the menstrual period the debility is so marked, so out of proportion to other symptoms, that one will, until he understands he is dealing with sycosis, be at a loss to account for it. Sycosis makes all plain.

During the period all the constant symptoms of the patient are more manifest and aggravated. During the menses there may be spasms of various muscles, particularly those of the neck, and those which move the head backward, and of the back. I have seen opisthotonus in several cases. Twitching in various parts; jerking of the head from side to side and backward are symptoms going to show the effect of this poison on the nervous system. And to make the condition worse is the ever-present anguish.

With the debility between the menstrual periods there is numbness all over the body, particularly of the upper and lower extremities; there is constant headache of varying kinds; pain, heat, and pulsation along the spine, and great tenderness of abdomen.

Sleeplessness is a distressing symptom, and from this the general condition is, of course, aggravated from want of proper rest. I am convinced that the majority of cases of so-called neurasthenia are of sycotic origin.

. The symptoms I have given as found in women are those of inherited sycosis, which is even worse than the original condition, and it is incumbent upon us as followers of Hahnemann, with his example before us, to continue to observe the various manifestations of this miasm, to the end that we may be able to treat our patients more intelligently.

I close with this hint: I am convinced that sycosis is the gonorrhœal poison plus psora.

---

Sycosis, which cannot be subdued by the vital powers alone, has never been regarded as a distinct species of chronic disease depending on an internal miasm; and it was supposed to be cured when the excrescences on the skin were destroyed, while no attention was paid to the source, which still continued to exist.—*The Organon*, § 79.

